# Special section on psychopharmacology trials in children and adolescents

**DOI:** 10.1186/1753-2000-2-35

**Published:** 2008-12-08

**Authors:** Joerg M Fegert

**Affiliations:** 1 Department of Child and Adolescent Psychiatry/Psychotherapy, University Hospital Ulm, Ulm, Germany

## Abstract

For the first time, we publish a special section of four related articles in the online, open-access journal Child and Adolescent Psychiatry and Mental Health.

## Editorial

This special issue is thematically focused on psychopharmacology, and offers readers the opportunity to examine recent developments in this area of research as described from different perspectives.

This section was the result of a conference organized by the Berlin Brandenburg Academy of Science, together with the European Academy, and held in Berlin, April 3^rd ^and 4^th^, 2008. During this conference, which was focused on ethical, methodological, legal, regulatory, and implementation issues in designing and conducting research in vulnerable populations, speakers from academia, industry, and government agencies discussed the most recent developments in pediatric pharmacology research, with special attention to the conditions determining the necessity and feasibility of conducting clinical trials in child and adolescent psychiatry.

Benedetto Vitiello (National Institute of Mental Health) informed about changes in the legal and regulatory framework in the U.S. and implications for clinical trials in minors. Birka Lehmann form the German Regulatory Agency informed about the new European framework and the role of European Medicines Agency (EMEA) and its committees in implementing the new regulations and generating more information about the safety and efficacy of pharmacological treatments in childhood. Philippe Auby, from Lundbeck Pharmaceuticals, discussed the new European pediatric research regulations from an industry perspective. Finally, academic researchers Jacinta Tan and Michael Kölch discussed ethical preconditions and pitfalls in child psychiatric pharmacological research in children and adolescents.

As a chair and discussant at that session I got the impression that these presentations altogether gave a very good overview of the current situation and both the advances and limitations in child psychopharmacotherapy research in the U.S. and Europe. The coordinated publication of these reports in CAPMH also offers an opportunity for readers from other parts of the world to comment on these issues by submitting letters and rapid communication articles.

The editorial team is open to suggestions from our readership and potential authors for other special sections so that we can invite authors to submit contributions relevant to one general theme under the coordination of a guest editor. We hope that the addition of thematically focused special sections to CAPMH will further enhance the attractiveness of the journal.

